# Listening to the Voices of Aboriginal and Torres Strait Islander Women in Regional and Remote Australia About Traumatic Brain Injury From Family Violence: A Qualitative Study

**DOI:** 10.1111/hex.14125

**Published:** 2024-07-20

**Authors:** Elaine Wills, Michelle Fitts

**Affiliations:** ^1^ Institute for Culture and Society Western Sydney University Parramatta New South Wales Australia; ^2^ Menzies School of Health Research Charles Darwin University Alice Springs Northern Territory Australia; ^3^ Australian Institute of Tropical Health and Medicine James Cook University Townsville Queensland Australia; ^4^ Centre for Alcohol Policy Research La Trobe University Victoria Bundoora Australia

**Keywords:** Aboriginal and Torres Strait Islander, concussion, family violence, remote, traumatic brain injury, women

## Abstract

**Introduction:**

Indigenous women experience high rates of family violence‐related head injuries. At present, lived experience accounts from Indigenous women are absent, which results in incomplete understandings and inadequate responses that have detrimental impacts on them and their families. The aim of this study was to gain insight into Indigenous women's personal and family perspectives regarding violence‐related traumatic brain injury (TBI), including impacts on life, as well as decision‐making processes about healthcare access and engagement.

**Methods:**

Purposeful sampling was used to complete semi‐structured interviews with 18 Indigenous women living in regional and remote Australia who had experienced TBI from family violence. The data from these interviews were augmented by data from interviews and focus groups with 28 community members, including family members or carers of Indigenous women living with TBI from family violence.

**Results:**

Three themes were conceptualised based on the data and research aims: interweaving of the past and the present—ways women experience brain injury; factors that inform decision‐making to access healthcare; and managing everyday changes that result from TBI from family violence. Indigenous women described living with a range of symptoms following repeated head injuries including problems with memory, cognition and concentration. A range of strategies to manage long‐term symptoms of TBI were used by Indigenous women and when they did seek healthcare, Indigenous women were required to navigate a range of barriers.

**Conclusions:**

The findings identify a range of gaps in healthcare and housing supports for Indigenous women with TBI from violence, highlighting the significant investment needed to develop responsive and appropriate pathways of care in regional and remote areas. A range of suggestions are discussed including development of a specialised workforce who can provide apppropriate follow‐up support, co‐designed concussion clinics and educational resources. TBI must also be a key aspect of policy and practice for services working with Indigenous women who have experienced violence to ensure appropriate responses are provided.

**Public or Patient Contribution:**

Indigenous women shared their views and experiences of TBI from family violence as well as decision‐making about accessing healthcare and managing TBI symptoms. As such, study participants provided public contributions to the research.

## 1 Introduction

Violence against women is a violation of human rights and a global public health problem [1]. In Australia, Aboriginal and Torres Strait Islander (hereafter, respectfully, Indigenous) women experience high rates of family violence [2]. Although reports vary, conservative estimates indicate that Indigenous women are 32 times more likely to be hospitalised due to violence compared to other women in Australia [2]. Family violence is the term used in this study and the preferred term in relation to framing family violence in the Indigenous context [3]. Unlike other terms, family violence reflects a holistic understanding that includes violence occurring within families and communities [3]. For Indigenous peoples, the use of violence within families and communities cannot be separated from the broader structural violence caused by intergenerational trauma arising from punitive historical and contemporary settler‐colonial policies and processes [4, 5].

Women who survive violence are at high risk of experiencing lasting impact on their brain [6]. Traumatic brain injury (TBI) can occur through a range of violent behaviours, including a direct blow to the head, a force transmitted from the body to the head, shaking of the body or the head, receiving hits to the head by objects or by external pressure that causes the blood vessels and/or air passages to close, depriving the brain of oxygen [7, 8, 9]. Mild oxygen deprivation with no loss of consciousness can result in a TBI [7, 10]. Given the high incidence of repeated violence, particularly in intimate partner relationships, women often experience multiple TBIs during the course of their lives [11, 12, 13, 14]. Studies involving women accessing violence‐related services found that 75% to 88% of women experience multiple TBIs related to family violence [13, 14]. Nonfatal strangulation is also increasing in the context of sexual violence [15, 16].

In studies involving women who have experienced repeated concussions from violence, the women tend to report psychosomatic symptoms, such as memory loss, confusion, difficulty following directions and difficulty concentrating and initiating self‐directed behaviour [14, 17]. Research has shown that an accumulation of concussion injuries can result in more severe symptoms [18]. Women may not immediately access healthcare following concussion for several reasons, including no visible signs of injury, low levels of knowledge and awareness of the lasting impacts of violence on the brain and fear of further violence [6, 19]. TBI and associated changes can also go undiagnosed by frontline professionals working with women who experience violence because of their lack of knowledge and training in the association between violence and TBI. Indigenous women may also be unaware of the severity of their symptoms [19, 20]. Despite Indigenous women experiencing high rates of TBI [21, 22], their views and perceptions are largely missing within the research literature that helps to inform decisions surrounding TBI healthcare [23, 24, 25].

The aim of this study was to gain insight into Indigenous women's perceptions of TBI, how they have managed their TBI symptoms and their decision‐making processes surrounding healthcare access and engagement. The information shared by Indigenous women will be critical for identifying how TBI services and support can be strengthened for Indigenous women, to improve their quality of life after TBI.

## 2 Materials and Methods

### 2.1 Design

A qualitative exploratory design was used by the authors in this study. Using this approach, the researchers were able to illuminate how a phenomenon that has received little research attention [23] is manifested, leading to the contribution of new knowledge in the area [26]. The study design was informed by the methodological literature on TBI and disability research with Indigenous peoples, which stressed the need for researcher reflexivity and nonhierarchical research relationships [27, 28]. Methodological decisions, including participant criteria, recruitment processes and resources required for the informed consent process were also informed by recurrent consultations with Elders, community members and women's groups [29].

### 2.2 Positionality

As researcher positioning is an essential component in the conduct of reflexive, ethical and quality research [30], the authors embedded within the research process describe their worldview and positionality. The first author is a Warumungu woman who has dedicated much of her career and studies to supporting Aboriginal women who have suffered family and sexual violence to access healthcare and other social justice needs. She cofacilitated interviews and focus groups in one region where the research was conducted. The second author is a white settler, who was raised on the unceded lands of the Bunurong people and now lives in Mparntwe. She was the project lead and facilitated interviews and focus groups across both regions involved in the project. She acknowledges the lasting and continued impacts of settler‐colonial systems and structures, including academia. Through decolonised research practices, she is committed to supporting the development of models of care that reflect the priorities of Indigenous women who sustain TBI from family violence. Both authors have had lived experiences of violence as children and adults.

### 2.3 Sampling and Participants

Purposeful and convenience sampling [31] was applied to recruit Indigenous women (aged 18+) who had experienced at least one TBI from family violence and had insight into their injury (Group 1). Following the guidance received during consultation with community members and other project stakeholders [32], Indigenous women were recruited through the support of community‐based organisations that had worked closely with Indigenous women who had experienced family violence. These organisations included Aboriginal community−controlled and mainstream legal and health services and women's groups. This sampling strategy has been used to connect with hard‐to‐reach groups elsewhere [33]. Key staff from these organisations and groups approached Indigenous women clients who attended their services. Once it was established that an Indigenous woman who met the criteria was interested in participating or hearing more about the project, the community‐based service staff member contacted the project lead (author M.F.) to schedule a convenient time and location for an interview. The second group of participants (Group 2) consisted of Indigenous women community members and Elders; they had diverse experiences and perspectives, including supporting or caring for family members living with TBI from family violence. Some participants in this second group also had work histories in family violence, advocacy, health or legal service sectors. They also represented diverse cultural and language groups. Although Group 2 participants were not recruited to collect information about their perspectives of lived experience of TBI from family violence, some Indigenous women in this group self‐reported that they had experienced injuries on their head, neck or face from family violence.

### 2.4 Data Collection

To support project promotion and data collection, TBI education sessions were delivered to women's groups and community‐based services by Indigenous facilitators from a national brain injury organisation [32]. This approach aligned with the principles of national guidelines for conducting research with Aboriginal and Torres Strait Islander peoples. The education sessions built sustainability and were intended to empower the wider community by disseminating knowledge surrounding TBI symptoms and management that had previously been unavailable [32, 34]. TBI education sessions were also intended to dismantle the stigma and shame associated with brain injury. Data collection commenced in March 2022 and finished in December 2023. All interviews and focus groups were conducted in English, with discussion between participants in some focus groups occuring in local language. Participation was voluntary, and all participants were provided with a plain‐English statement about the research as well as the project flipchart and signed consent forms. Participants had the option to have their interview or focus group digitally recorded or for written notes to be recorded by a research team member.

The research team met with Indigenous women from Group 1 on at least two or three occasions. Multiple meetings helped the research team to develop rapport with each participant and manage participant fatigue. For Indigenous women in Group 1, topics explored included the following:

- understanding of a head injury,
- a lifetime history of head injury,
- symptoms of head injury and strategies to manage them,
- why they did or did not get medical treatment for the head injury,
- experience in accessing hospital care,
- opportunities and challenges of healthcare access and other support services,
- recommendations for improved services and support systems.

Although a topic list guided the process, Indigenous women were encouraged to talk freely and could tell their stories in their own way. The flexible interview approach allowed for the exploration of additional issues raised by Indigenous women [35]. For participants in Group 2, the questions covered were as follows:

- the types of support and services available to Indigenous women,
- enablers and barriers women experience when accessing services,
- challenges for service providers supporting Indigenous women,
- suggestions for reform,
- improvements for policy and service delivery.

Participants from both groups nominated their preferred location to complete their interviews and focus groups. These included the office of the service provider, the local library, an outdoor area at a botanical garden and the workplaces of participants. Most interviews and focus groups were conducted jointly by an Aboriginal researcher and a non‐Indigenous researcher. Paired interviewing allowed the research team members to reflect on, discuss, compare and make meaning of information shared in interviews, as well as identify the strengths and weaknesses of the interview process and environment [36, 37].

### 2.5 Data Analysis

Interviews and focus groups were audio‐recorded and transcribed verbatim by a professional transcription service. Written notes from each interview and focus group were also transcribed into a Microsoft Word document and assigned a pseudonym. Each transcript was reviewed for accuracy and then offered to participants for their review and comment. Anonymous transcripts were subsequently analysed using reflective thematic analysis as described by Braun and Clarke [38]. The process was inductive, as the data were not being fitted into a pre‐existing framework and this allowed for themes to emerge from patterns of meaning and unexpected perceptions. The first phase of analysis involved reading and re‐reading the transcripts and listening to audio recordings to increase familiarity with the data. Once familiar, the authors made handwritten notes on the transcripts to generate an initial list of ideas and identify early concepts for coding. All transcripts were managed using qualitative data analysis NVivo V.12 (QSR International Pty Ltd). Initial ideas were discussed between the authors and, when an agreement was reached, codes were organised into groups and initial themes were developed. The next phase involved reviewing the data extracts coded under each theme and deciding whether or not they fit within the theme and if they formed a sound and logical pattern. Independently, each coder completed this process before reaching a consensus and amending NVivo. This process was repeated; the researchers re‐read the transcripts and coded any additional material to the new codes. In the next phase, each theme was renamed, defined and summarised. A selection of community members from the two regions were consulted by the research team to contribute to data analysis and to ensure the accuracy of the quotes and naming of the themes. The knowledge from these meetings was incorporated into the coding and final naming of themes. The authors ensured that each theme had clear parameters and fitted into the broader narrative of the complete data set. All extracts were checked for accuracy. The final phase involved writing up the findings to reflect the interpretation of the data.

### 2.6 Artwork

**Figure 1:**
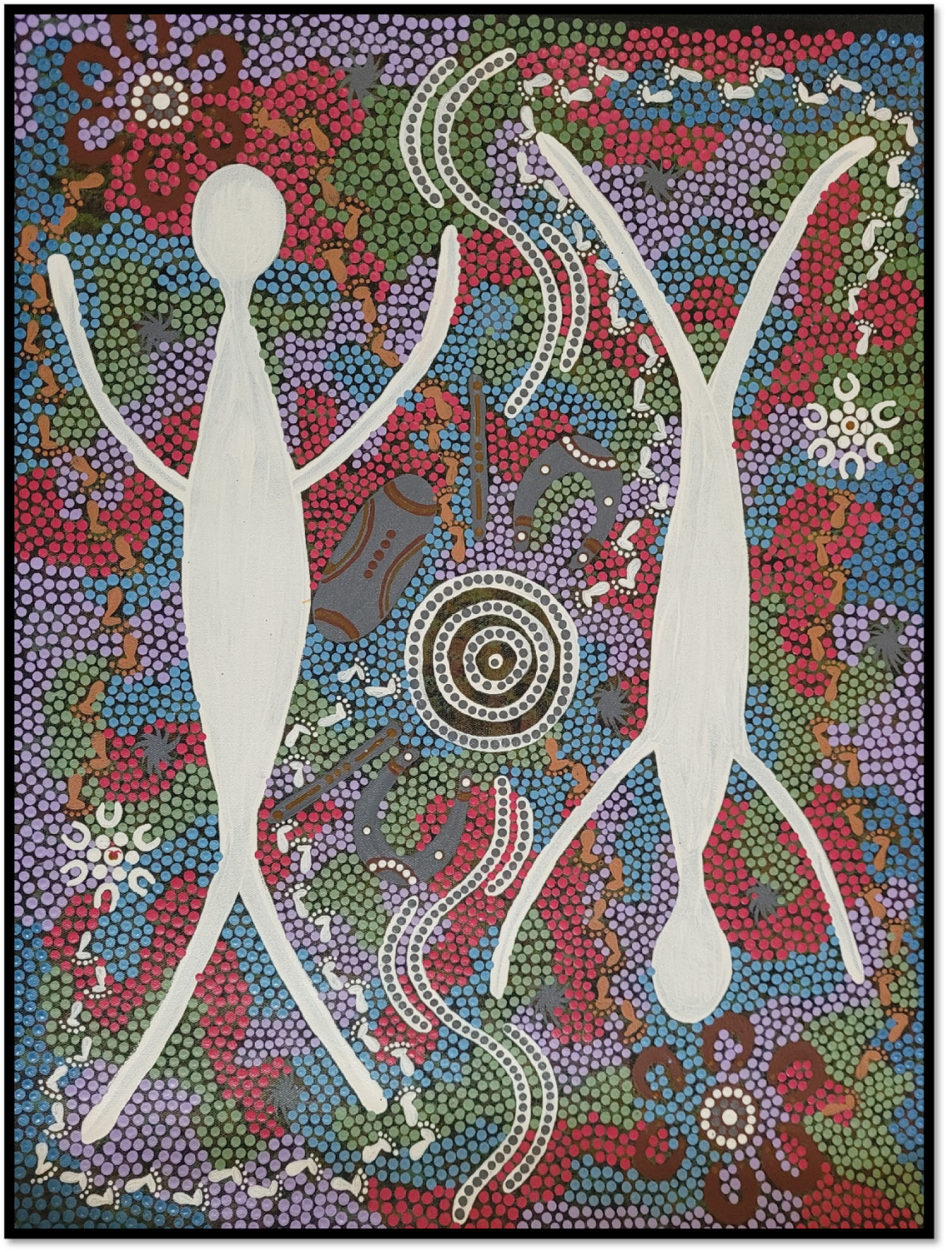
Shirleen Nampajinpa Campbell, *Journey Home After Brain Injury*, 2021.

**Figure 2:**
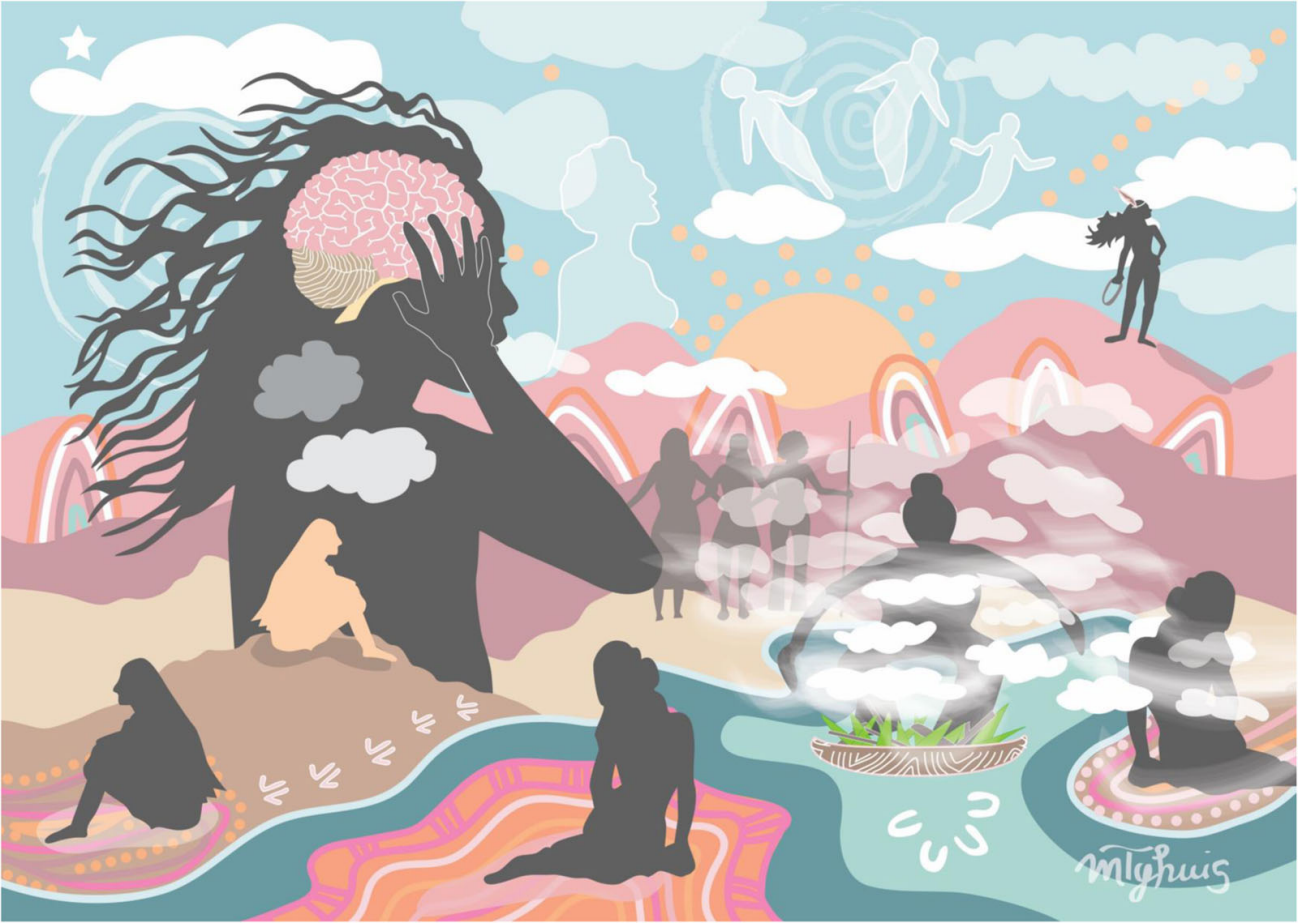
Michelle Tyhuis, *Healing Her Beautiful Mind*, 2023.

The project commissioned artwork to promote the purpose and intent of the research in a manner that demonstrated cultural respect and understanding (see Figure 1). Artwork was also commissioned to reflect the key experiences of Indigenous women who participated in the study (see Figure 2).

#### 2.6.1 Journey Home After Brain Injury

Shirleen Nampajinpa Campbell is a proud Anmatyerre and Arrernte woman and a fifth‐generation resident of Alice Springs Town Camp, Lhenpe Artnwe or Hoppy's Camp. She is also the coordinator of the Tangentyere Women's Family Safety Group (TWFSG), which gives women in the camp a voice and action against family and domestic violence. The art (Figure 1) highlights the role that female ancestors play in guiding women through their journey throughout each generation. Women will always know where they belong and what makes up their identity through language, song, dance and connection to the land. Women are the nurturers, teachers, guides and storytellers because they play a big part in keeping the culture healthy and strong. Service providers who are Aboriginal women can draw on their experiences to make life easier for Aboriginal women when working with them. This means that everyone can be supported and looked after. The white dots with footprints represent the service providers walking in without cultural knowledge. Through deep listening to our lore female Elders, service providers are taught to practice in a way that is safe for women and girls in the community and safe for them. Learning the knowledge of Aboriginal culture, the system and structure means that service providers can share in two‐way learning and deep listening as tools in all areas when dealing with women after head injury. The two large white images represent ancestors, who guide the journey. Six young women are sitting and waiting for that support and sharing their stories to help other women and girls.

#### 2.6.2 Healing Her Beautiful Mind

This artwork (Figure 2), created by Michelle Tyhuis, a Torres Strait Islander woman from Meriam Mir (Erub Darnley) Island, is imbued with a strong cultural respect for the wisdom of ancestors and symbolism. The woman with her hands to her head represents the distress and vulnerability that can be generated with repeated TBIs and was reflected in the narratives of Indigenous women who participated in the study. The woman on the hill sitting backward represents a past self, with the person she used to be changed from repeated TBIs. The woman sitting inside the body of the woman with her hands to her head under the clouds represents loneliness, isolation and managing days of worry and sadness as she lives with the cognitive, psychological and behavioural symptoms of TBI. The barriers to accessing support services and healthcare, being lost both within herself and the community, are reflected by the patterns under the woman at the bottom centre of the artwork. Recovery for Indigenous women involves family support, community connections and healing on country, and this is reflected in the patterns under the woman on the bottom right of the artwork, as well as the woman in the smoking ceremony. The woman at the mountain, at the top of the artwork, honours Indigenous women who follow their own pathway of recovery, self‐belief and strength.

### 2.7 Ethics Approval

This study was approved by the Central Australian Human Research Ethics Committee (CA‐21‐4160), Townsville Hospital and Health Service Human Research Ethics Committee (HREC/QTHS/85271 and HREC/QTHS/88044) and Western Sydney University Human Research Ethics Committee (H14646). Guidelines for conducting research with Aboriginal and Torres Strait Islander peoples were followed at each of the project locations [34].

## 3 Findings

Eighteen interviews with Indigenous women who had experienced TBI from family violence participated in the project. Group 1 participants were aged between 26 and 68 years of age at the time of the interview, with the average age being 40 years. A further 28 women participated from women's groups. Each theme was informed by the voices of Indigenous women from both regions. All participants reported that this was the first time they had had an in‐depth discussion on their experiences of head injuries from family violence. As presented in Table 1, three themes were conceptualised based on the data and research aims: (1) interweaving of the past and the present—ways women sustain brain injury; (2) factors that inform decision‐making to access healthcare; and (3) managing everyday changes after TBI from family violence.

**Table 1:** Overall themes, subthemes and examples across perspectives.

Themes	Subthemes	Example codes
Theme 1. Interweaving of the past and the present—Ways women sustain brain injury	Traumatic brain injury from family violence	Injury occurrence; injury characteristics; characteristics of the person who used violence; nonfatal strangulation
	Other ways harm and injuries occur to the brain	Other forms of traumatic brain injury; intergenerational trauma; psychological trauma; wrong skin relationships
Theme 2. Factors that inform decision‐making processes to access healthcare	Characteristics of the injury	Visible injuries; loss of consciousness; symptom experience
	Situational factors	Coercive control; risks of further violence; housing circumstances
	Previous healthcare experiences	Accessibility of emergency department and healthcare; hospital and healthcare environment; medical care, treatment and education; after discharge experiences
Theme 3. Everyday changes from traumatic brain injury from family violence	Ways women experience traumatic brain injury	Cognitive symptoms; autobiographical memory loss; changes in roles or activities; change in autonomy; changes to taste, smell and hearing
	Strategies to manage everyday symptoms	Personal strategies; family and community support and strategies; community‐based organisation support

### 3.1 Theme 1: Interweaving of the Past and the Present—Ways Women Sustain Brain Injury

In this theme, Indigenous women shared information about brain injuries resulting from family violence, as well as other perspectives on brain injury. The subthemes include TBI from family violence and other ways in which harm and injuries occur to the brain for Indigenous women.

#### 3.1.1 TBI From Family Violence

Within the narratives of Indigenous women with lived experience, head injuries from family violence were not isolated experiences; they were repetitive and occurred over prolonged periods. For example, some Indigenous women reported hits to the head every 2 weeks from their partner while they were in a relationship where there was violence. Partners included Indigenous men as well as non‐Indigenous men. Other Indigenous women reported they had ‘lost count’ of the number of violence‐related head injuries they had experienced. A range of objects were used by the men who used violence, including fists, axes, knives, steel or wooden poles. As Emily explained:

Didn't look after us, speak rubbish way to me in public, violent to me. Hits me when I'm sleeping and tells me what he is going to do. Hit me with his fist anywhere on my body, unconscious many times. In hospital a lot, one night, police took me to the refuge, he might kill me.

(Emily, Region 1)

As highlighted by Darcy's experience, household items such as cups, glass bottles, cans and drinking glasses were also used to cause injury: ‘He hit me with a bottle of rum, I blacked out’ (Darcy, Region 1). Another frequently reported experience of Indigenous women was being dragged by their hair and having their head hit onto the ground or floor. At the time of sustaining head injuries, Indigenous women described other facial injuries, including fractured jaws, broken teeth and injuries to their eyes and ears. Rowena shared the following experience: ‘The big punches, I was like a punching bag, eyes, cheeks, nose, ears’ (Rowena, Region 2).

Stab wounds, swelling and bruising to their head or other parts of their body were also recounted within the stories shared by Indigenous women. Nonfatal strangulation, or what was commonly referred to as choking, was sometimes experienced by Indigenous women, particularly those aged under 30. Indigenous women and Elders also shared that violence‐related head injuries can also come from other women:

Fighting between women, jealously, use scissors, bald patches on head from being stabbed with scissors, jealously. Use small scissors, stabs to head, pulling hair out leaving bald patches, hitting head, head injuries.

(Community group, Region 1)

#### 3.1.2 Other Ways Harm and Injuries Occur to the Brain

TBI from family violence was considered one of many ways lasting harm to the brain can occur for Indigenous women. Sustaining a TBI as a child through a motor vehicle crash was an experience frequently mentioned by Indigenous women with lived experience of TBI. Enduring complex psychological trauma from the physical violence experienced by Indigenous women was viewed as having lasting impacts on brain function and generating similar emotional symptoms similar to TBI:

It is mental health, trauma or head injury or a combination of all those things. I know the impact of the physical trauma from my partner. But it was the mental trauma as well. That also changes your brain, how it is wired and how it works.

(Community member—women's group, Region 1)

Women with possible head injuries, they change directions with what they're talking about. I don't know if it's memory. It's probably trauma, both physical and psychological trauma.

(Community member—women's group, Region 1)

Witnessing violence during childhood and as an adult was also discussed as having a long‐term effect on the brain. Traumatic events included the death of partners and family from car accidents and suicide. Four Indigenous women with lived experience mentioned that their mothers had experienced physical violence when they were in utero, and this may have had an impact on their brain development. Another example was from a women's group in Region 1, who raised that intergenerational trauma can be transmitted through genetics. Women's groups also frequently mentioned that the destructive practices of settler colonisation to cultural systems, including the kinship system, have also affected brain health:

Right skin, then was happy, that relationship was forever. It's terrible, wrong skin, different languages, skin groups, old ways of marrying your own tribe. Wrong skin, causes damage to heads, blood is poisoned.

(Community member—women's group, Region 1)

### 3.2 Theme 2: Factors That Inform Decision‐Making to Access Healthcare

A range of factors were considered by Indigenous women at times of decision‐making to access healthcare following TBI from family violence. This theme includes the following subthemes: characteristics of the injury, situational factors and previous healthcare experiences. When making decisions about access to healthcare, a combination of these factors influences the decision‐making process for Indigenous women.

#### 3.2.1 Characteristics of the Injury

Accessing healthcare was a carefully calculated decision‐making process in which Indigenous women considered injury‐related factors including: if they lost consciousness during the violence; if there was a visible wound or blood; if they were able to manage symptoms and pain; and if they experienced other injuries in the head region (e.g., broken teeth, injury to their jaw). When Indigenous women did not experience or recall a loss of consciousness, or if there was no visible blood, they usually self‐managed any symptoms and did not usually access hospital care:

He come from behind me. I was standing in the kitchen making dinner. Then whack. I made sure I didn't fall to the ground, so I didn't go down. I held on to the bench. He didn't knock me out, I saw stars. There was no blood. I had a headache for a few days.

(Marlee, Region 2)

Can get jealous, called and text me. Then one night he hit me. There was no hospital, no blood, bleeding, no one would have thought there was domestic violence that happened to me. Didn't think it was serious enough to go. On the ground, passed out, woke up, couldn't remember what happened really.

(Cathy, Region 2)

#### 3.2.2 Situational Factors

Decision‐making about access to healthcare also involved consideration of the situational factors surrounding the injury, including the potential for further physical violence from their partner or family and the location where the injury occurred, as well as the potential involvement of or referral to government systems such as child protection:

He hit me at the accommodation we were staying. We had [our son] in the nursery at the time. He was only a few days old. We were in his Country. This wasn't my country. He had lived there before, but not me. I didn't know anyone. All my family was back home. I didn't feel safe to report what had happened.

(Kirra, Region 1)

Arguments with partners' family members, who directed anger at Indigenous women for accessing healthcare, reporting family violence to authorities and managing coercive control by the person who used violence:

He was living with his mum. She said, ‘Why did you put him away? You should teach him not to do this to me. I don't like to be the one in pain and injured.’

(Beatrice, Region 1)

He says, ‘If you press charges, then [there] must have someone else you are seeing and you want to be with them. Show me that you are not with someone else by not pressing charges.’

(Amanda, Region 2)

#### 3.2.3 Previous Healthcare Experiences

Mixed experiences of hospital care were reported by Indigenous women. Extended waiting times in emergency departments (EDs) resulted in them leaving the ED without having been assessed for their injuries. Indigenous women who received care said that their visible injuries, including lacerations and bruising, were attended to thoroughly. In relation to TBI, some Indigenous women stated that they received a computed tomography (CT) scan, while others were unable to recall, and some stated they did not receive a scan for their head injury. Indigenous women who recalled being advised by medical professionals that their CT scan was ‘clear’ of serious injury questioned how this could have been possible because of the lasting everyday impacts they noticed in themselves after their ED presentation. Some Indigenous women spoke about the hospital environment being loud and busy, contributing to feelings of frustration and confusion.

Indigenous women who received medical assessment stated that they did receive contact with a health professional and referrals to community‐based services, including legal, family‐violence and victims‐of‐crime services, but there was no one service specifically for TBI. Some Indigenous women did receive follow‐up from local health and community‐based services, but some Indigenous community members mentioned that none of the staff working there had training in TBI:

I didn't always go hospital. Sometimes you can't go, you get held back. Then you go. Nothing from the hospital, once you leave, you can't contact them again. You call the main number and they told me to contact my local doctor. Why did I go? No one followed‐up with me.

(Lila, Region 2)

They will follow‐up but no one working there with this specialised training in the area [of brain injury].

(Community Elder, Region 2)

As illustrated in Figure 1, Indigenous women sometimes sought treatment through cultural pathways, such as traditional healers and medicine, and smoking for cleansing and treating sickness: ‘If it's not dealt with properly, go and see a witch doctor first, then go to the clinical doctor—witch doctor will refer to other people, do clinical and cultural medicine’ (Community elder, Region 2).

### 3.3 Theme 3: Managing Everyday Changes From TBI From Family Violence

Indigenous women described the cognitive, psychological and behavioural symptoms they managed in their everyday lives following their injuries and the ways they managed these changes. Subthemes include ways women experience TBI and strategies to manage everyday symptoms.

#### 3.3.1 Ways Women Experience TBI

Changes to memory and executive functioning were the symptoms identified most often by both Indigenous women, who noticed these changes in themselves, as well as those who noticed them in friends and family members who had experienced long‐term violence in current or previous intimate partner relationships. Changed cognitive functioning was connected to missing appointments with services, not remembering whether or not they had attended an appointment, inability to locate critical documents and information that had been stored for safety and failing to recall if they had prepared and eaten meals during the day. A strong source of frustration for Indigenous women was their autobiographical memory loss that disrupted their access to self‐history and important cultural stories shared and passed on to them:

Black out, now suffering from memory loss, like finding hard to be telling a yarn. These are stories that have happened to me. But I can't remember it.

(Coral, Region 2)

I notice short term memory problems in myself. I just blank out, I forget things. Sometimes someone is telling me something and then I feel lost. Other things, I put something somewhere, like, book, keys, phone. If I can t see it, I forget where I put it. I have troubles keeping focused on one thing. I thought this was normal, it was old age. My daughter said, it s not okay and worries for me.

(Denise, Region 1)

Start talking about something then stops then goes back after, ‘I was speaking about that’. Short‐spaced memory, ‘forgot about that’.

(Community Elder, Region 2)

Indigenous women described this as ‘losing the words or having the words disappear’ or feeling like ‘my brain went blank’. Other changes Indigenous women and family members noticed included changes in balance and coordination:

Any family ask me, ‘you been drinking?’ Any little thing I do. It goes like that [participant walked in the interview], walking really stiff and I keep tripping. One little slide on the path, I'm so sloppy, I'm not even walking properly. I have to stay home.

(Katherine, Region 1)

Changes in the person, memory, being forgetful, slurred speech, balances challenges, words jumbled, words are unclear.

(Community Elder, Region 1)

Changes in personality and mood were mentioned more prominently by women's group members who reflected that their family member or friend was not who they once were. They mentioned heightened levels of anger and aggression or changes from being shy to being loud: ‘Notice she becomes silent, prefers to be alone, forget things, speaking is different, mood swings. She used to be quiet and gentle. She now gets really angry’ (Community group member, Region 1).

These executive functioning and personality changes were identified by both Indigenous women themselves, after they had experienced multiple TBIs, and community members. They described lasting impacts displayed by Indigenous women they knew who were aged over 40. Both participant groups mentioned that they perceived there to be an increase in Indigenous women who displayed signs of early‐onset dementia:

We have noticed in the community, the number of women who have had early onset dementia, it has increased. We know in our minds, it could have come from all the violence.

(Community member interview, Region 2)

#### 3.3.2 Strategies to Manage Everyday Symptoms

Tactile and mindfulness activities, such as painting, listening to meditation music and completing puzzles were among the activities Indigenous women used in their daily lives to strengthen memory and manage anxiety. Family members played an important role in supporting Indigenous women with everyday tasks, such as shopping, paying bills, washing clothes and cooking, which could be impacted by TBI symptoms causing difficulties with memory and information retention. As Pat explains:

See, I can't even do my own shopping. Someone from the family talks to me on the phone when I'm at the shop so I don't forget. But if they are not on the phone or someone isn't with me, then I forget to get it. Sometimes my daughter or grandchildren will take a photo and send to me, ‘Don't forget that main ones, washing, meat.’

(Pat, Region 1)

Indigenous women managed TBI symptoms and feelings of vulnerability and maintained a sense of personal safety in diverse ways. Isolating themselves from family, friends and social circles to manage headaches and minimise the potential for arguments with family was a recurrent strategy in stories shared by Indigenous women, as Emily and Evelyn explain:

Sometimes I tell my family to shut the hell up. That's, if they're too many people talking. Then I have to leave, I want to be on my own. It is painful, gives me headaches, I can't deal with the buildup of pain. There are too many people talking in one space.

(Emily, Region 1)

Grandson is worrying for me. I used to do things all the time. Now I get scared from everything, you know. I am wanting to be by myself. Sometimes I go to the park across the road from where my daughter lives. The park is for somewhere quiet. I sit there.

(Evelyn, Region 2)

Other ways in which Indigenous women managed personal safety included organising meetings with friends when frequenting public areas (e.g., supermarket or social club) where there were people, including in some cases, the person or people who had used violence against them. Male family members also played an important role in maintaining a sense of safety for Indigenous women; they organised to live with or regularly visited the homes of their brothers and uncles. A sense of belonging and social connectedness through church and women's groups was also critical for some Indigenous women: ‘We go down each Tuesday, have a cup for tea and lunch, there is no judgement and it's a woman's only space’ (Margaret, Region 2).

Indigenous women shared stories in which they felt some community‐based organisations or government services did not fully understand and respond to the symptoms of TBI and their current circumstances. For example, some services suggested that Indigenous women use strategies to manage memory‐retention challenges, including writing down information, using wall calendars and saving meeting times in electronic calendars on mobile phones. These strategies were unhelpful for some Indigenous women, who recalled that their phone could be taken by a partner, could not always be charged due to limited access to electricity or could be misplaced. Indigenous women stated that, even if they did have the reminder in their phone, they often did not have the transport and financial resources to attend health appointments. Homelessness was experienced by most Indigenous women interviewed, with some describing cycles of living with family members who were already managing overcrowded homes as well as in sleeping in hostels, women's shelters or public spaces. As Denise stated, not having a home or dedicated space of her own created difficulties with fulfilling obligations and conditions to maintain access to support services, including social support:

I can't keep going to the Newstart [social security payments, now Job Seeker Payment]. I keep forgetting. I can't remember what you're telling me. See I can't. I tell them that. They say, ‘Write it down’. I say, ‘I lose the paper’. They tell me to save information in my phone or have a calendar. I am staying with my daughter.

(Denise, Region 1)

## 4 Discussion

Broadly consistent with other literature on TBI from family violence [13, 14, 25, 39, 40], Indigenous women in this study described a range of cognitive, psychological, behavioural and physical changes they had noticed in themselves or observed in other women who were important to them, following repeated head injuries from violence. The well‐documented effects of settler colonisation, including the destruction of kinship structures and wrong‐skin relationships, as well as psychological trauma from witnessing and experiencing violence [4, 41], were also considered by Indigenous women in this study to have lasting impacts on brain health. Frequently reported barriers to accessing medical assessment and treatment for TBI from family violence were identified, including fear of retaliation from their partners or extended family, lack of adequate transport or fear related to involvement of government systems such as child protection [4, 42, 43]. Characteristics of the injury also appeared to play a role in decision‐making to seek healthcare, including recalling a loss of consciousness and injury visibility.

At the ED, uncertainty about what assessments and medical investigations were completed, variability in approaches to TBI education and lack of postdischarge follow‐up or meaningful connection with community‐based organisations reflected early‐documented limitations of healthcare and service responses to women with TBI from family violence [25, 44]. Although commonly used strategies to manage TBI‐related symptoms can be beneficial for some women who have sustained TBI from family violence, for other Indigenous women these strategies were unable to be realistically implemented in everyday life. For example, homelessness is linked to family violence and undermines the capacity of Indigenous women to seek medical care and treatment for TBI and implement strategies to manage everyday TBI symptoms [45]. Access to safe housing is a foundational component required for long‐term recovery and prevention of further disability generated by violence for Indigenous women.

Informal support from friends and family, help from women's groups and traditional forms of healing are important for Indigenous women experiencing family violence [46]. For some Indigenous women, these networks were critical to feel safe and supported. Similar to other studies, Indigenous women in this study employed physical isolation and social withdrawal (e.g., from family and social gatherings) to manage and maintain the highest level of safety possible, as well as to minimise the risk of being identified as having a brain injury [17]. Communication troubles from a brain injury can lead to feelings of frustration and not feeling like the person they used to be [47]. Yarning, sharing and recalling stories are vital to feelings of identity, inclusion in the community and self‐worth.

### 4.1 Implications of the Study for Health Services

Improving opportunities to minimise the impacts of TBI is possible, but it demands significant investment in research, education and services to develop processes of healing and rehabilitation tailored to Indigenous women, families and communities. Adequately funded resourcing of local services that provide connection, including women's groups and traditional healing practices in regional and remote towns and communities, is important for supporting Indigenous women living with the impacts of family violence. Traditional healing practices have been shown to establish and strengthen relationships in response to trauma [41]. Greater investment in brain injury services in regional and remote areas, such as concussion clinics with multidisciplinary teams [48], is needed to address the noticeable absence of brain injury supports available outside of major cities. Future research could codesign guidelines and development of concussion clinic spaces in regional and remote Australia. A coordinated care pathway after discharge from a hospital or community clinic, including follow‐up support from a specialised workforce, such as Aboriginal social workers or Aboriginal allied health workers with training in TBI and violence, is also needed [44]. Traditional healing, healthcare and concussion clinics must be augmented by access to immediate crisis and short‐term housing and other support systems that consider the complexities of both TBI and family violence, to ensure Indigenous women can meaningfully lead and engage in their rehabilitation. Short‐ and long‐term housing options would minimise risks of further head injury during recovery for Indigenous women.

There is an urgent need for community‐based services to develop and implement TBI‐informed policies and practices, mobilising the national plan recommendations of including TBI in service responses to family violence [49, 50]. TBI prescreening and screening within different service sectors (e.g., primary healthcare, dental care, women's shelters, legal services, and tertiary healthcare) as well as the inclusion of TBI‐informed community‐based service policies could help identify TBI symptoms and facilitate referral pathways to support Indigenous women [19, 51, 52]. Although conversations surrounding TBI can generate a range of emotions, including anger and shame, earlier work in the United States has shown that for some non‐Indigenous women identification and diagnosis of TBI can generate ‘diagnostic‐relief’ by providing an explanation for symptoms and changes they were experiencing [53]. Strengthening TBI knowledge and skills of the frontline workforce is required to ensure there is awareness that family violence can generate forms of disability [54]. Training would also build workforce confidence to screen for TBI and respond appropriately and is critical to minimising the likelihood that TBI behaviours and symptoms displayed are not misunderstood as being noncompliant and impatient [55].

Providing communities with resources to develop locally led education to grow TBI knowledge and skills for Indigenous women, families and community is needed. As community knowledge surrounding TBI is low [32], such knowledge could help validate and legitimate the challenges Indigenous women have faced through disability, generated by violence but not fully understood [17]. Tailored information about concussion should be provided to Indigenous women who have experienced TBI and family members, including key aspects of the injury and treatment that they may be unaware of, such as the long‐term cellular‐level changes that occur to the brain with repeated head injuries from violence; as well as terminology surrounding medical procedures and assessments for TBI (e.g., CT scan). Building a deeper understanding of TBI could support Indigenous women to negotiate these changes with more self‐understanding, kindness and compassion as well as help them to implement positive health actions for recovery and enable them to make informed health decisions [56]. For prevention, there are also growing calls from community leaders for codesigning concussion education with young people that can be implemented in schools [43].

The suggestions made here must also be contextualised within the social–cultural and ethical–legal environments. The intersectionality of family violence, disability and TBI has the ability to make Indigenous women more vulnerable to settler‐colonial systems and structures. For example, Boyle et al. [57] have also shown that brain injury screening can be used to undermine the ability of women to fulfil their role as a parent. Mothers with disability continue to experience stigmatisation and struggle to achieve equal rights and full participation in society.

### 4.2 Strengths and Limitations

This study included Indigenous women from two regions in Australia only. However, these regions are diverse and include women from different Aboriginal and Torres Strait Islander cultural groups. This means that the findings may apply to Aboriginal and Torres Strait Islander cultural groups more broadly. The research team connected with Indigenous women who were recruited through convenience sampling and connected with at least one community‐based organisation. Their experiences and insights into TBI may not be reflective of other Indigenous women who are connected with no services. As Indigenous women in the study described prior experiences of nonfatal strangulation and trauma, as well as mental health conditions, the psychological, behavioural and cognitive symptoms they described may have stemmed from factors outside of TBI, including anoxic brain injury. Many Indigenous women who participated in the study could not recall the number of TBIs they had experienced from family violence. Future research could consider the inclusion of medical record reviews. For some Indigenous women, English was not their first language, and this may have affected the information collected during interviews. No Indigenous women consented to have an Aboriginal interpreter or translator present at interviews. To ensure the meaning of interview transcripts was interpreted accurately, they were checked with Indigenous women. Early coding of the data was also discussed with some participants and project advisors.

## 5 Conclusion

To the knowledge of the authors, this is one of the first studies to focus on developing a deeper understanding of the experience of TBI from family violence for Indigenous women in regional and remote Australia and how they navigate everyday changes. Indigenous women in this study often started to experience TBI during their childhood and youth and continued to experience repeated TBIs from family violence as adults. Other forms of lasting harm to brain health were also identified by Indigenous women, including psychological trauma. While strategies to manage TBI symptoms can support some Indigenous women to manage everyday TBI symptoms, complex housing and social environments meant that it was often difficult for other women to implement them effectively. With a lack of brain injury services in regional and remote Australia, future research is needed to strengthen the acute care and community‐based organisation responses, which are not currently tailored to the intersectionality of TBI from family violence, as well as develop and implement a holistic model of care for TBI that nurtures pathways of support and empowers Indigenous women to flourish and thrive.

## Author Contributions

**Elaine Wills:** methodology, writing – original draft, formal analysis, investigation. **Michelle Fitts:** conceptualisation, funding acquisition, methodology, writing – original draft, writing – review and editing, formal analysis, project administration, validation, investigation.

## Disclosure

The content is solely the responsibility of the authors and does not necessarily represent the official views of the Australian Research Council.

## Conflicts of Interest

The authors declare no conflicts of interest.

## Data Availability

The authors have nothing to report.
